# A fast sample shuttle to couple high and low magnetic fields and applications in high-resolution relaxometry

**DOI:** 10.5194/mr-6-229-2025

**Published:** 2025-09-03

**Authors:** Jorge A. Villanueva-Garibay, Andreas Tilch, Ana Paula Aguilar Alva, Guillaume Bouvignies, Frank Engelke, Fabien Ferrage, Agnes Glémot, Ulric B. le Paige, Giulia Licciardi, Claudio Luchinat, Giacomo Parigi, Philippe Pelupessy, Enrico Ravera, Alessandro Ruda, Lucas Siemons, Olof Stenström, Jean-Max Tyburn

**Affiliations:** 1 Bruker BioSpin GmbH, Rudolf-Plank-Str. 23, 76275 Ettlingen, Germany; 2 Chimie Physique et Chimie du Vivant, CPCV, Département de chimie, École normale supérieure, PSL University, Sorbonne Université, CNRS, 75005 Paris, France; 3 Bruker BioSpin AG, Industriestrasse 26, 8117 Fällanden, Switzerland; 4 Department of Chemistry “Ugo Schiff”, University of Florence, via della Lastruccia 3, Sesto Fiorentino, 50019, Italy; 5 Magnetic Resonance Center (CERM), University of Florence, via Sacconi 6, Sesto Fiorentino, 50019, Italy; 6 Consorzio Interuniversitario Risonanze Magnetiche MetalloProteine (CIRMMP), via Sacconi 6, Sesto Fiorentino, 50019, Italy; 7 Bruker BioSpin, 34 rue de l'Industrie BP 10002, Cedex, 67166 Wissembourg, France

## Abstract

Combining high-resolution high-field nuclear magnetic resonance (NMR) with an evolution of spin systems at a low magnetic field offers many opportunities for the investigation of molecular motions and hyperpolarization and the exploration of field-dependent spin dynamics. Fast and reproducible transfer between high and low fields is required to minimize polarization losses due to longitudinal relaxation. Here, we introduce a new design of a sample shuttle that achieves remarkably high speeds ( $v_{\max}$ ∼ 27 m s^−1^). This hybrid pneumatic–mechanical apparatus is compatible with conventional probes at the high-field center. We show applications in water relaxometry in solutions of paramagnetic ions, high-resolution proton relaxometry of a small molecule, and sample shuttling of a solution of a 42 kDa protein. Importantly, this fast sample shuttle (FSS) system is narrow, with a diameter of d = 6 mm for the sample shuttle container based on a standard 5 mm outer diameter glass tube, which should allow near access to the sample for magnetic manipulation at a low field.

## 1 Introduction

High-resolution nuclear magnetic resonance (NMR) is preferably performed at high magnetic fields, with applications in chemistry, structural biology, material sciences or metabolomics. On the other hand, many types of NMR experiments require or benefit from access to low magnetic fields. Relaxometry probes molecular motions by quantifying relaxation over a broad range of magnetic fields typically down to 100 µ T (Kimmich and Anoardo, 2004). Many hyperpolarization methods can also benefit from low magnetic fields, such as chemically induced dynamic nuclear polarization (CIDNP) (Grosse et al., 1999; Li et al., 2023), parahydrogen-induced polarization (Pravdivtsev et al., 2015), or Overhauser DNP (Ravera et al., 2016; Reese et al., 2009) or from cycling between high and low magnetic fields (Ivanov et al., 2014). The relaxation rates of long-lived states (Carravetta et al., 2004), long-lived coherences (Pileio et al., 2009; Sarkar et al., 2011), and multiple-quantum coherences under chemical exchange (Cousin et al., 2016b) are diminished at a low magnetic fields that reduce the chemical-shift interaction. Zero- and ultra-low-field methods require even lower magnetic fields, in the nT range, to make the nuclear Zeeman interactions smaller than scalar couplings (Barskiy et al., 2024). The analytical power of high-resolution high-field NMR can be combined with all these approaches if an apparatus can transfer the sample between the magnetic center of a high-field spectrometer and a position of low field, typically over a distance on the order of a few centimeters up to ca. 1 m.

Physically moving the sample between high- and low-field positions takes time, usually tens to hundreds of milliseconds. Such transfer times are particularly limiting for applications in molecular systems with fast longitudinal relaxation, such as nuclear spins in paramagnetic complexes, which relax fast at all magnetic fields, or in macromolecules, where fast longitudinal relaxation is encountered at low magnetic fields. Coupling high and low magnetic fields with such systems requires moving the sample as fast as possible. It follows that sample shuttles are preferable to probe-shuttle systems (Grosse et al., 1999; Victor et al., 2004) as the inertia of the probe makes the displacement of the sample necessarily slow. Sample shuttles are moved by either a mechanical system, where the action of a motor is transferred to the sample by a cord (Miéville et al., 2011; Pileio et al., 2010); a belt (Chou et al., 2012; Redfield, 2012), a rack (Kiryutin et al., 2016); or a pneumatic system (Charlier et al., 2013; Kerwood and Bolton, 1987; Redfield, 2003; Reese et al., 2008). Pneumatic systems take advantage of moving a sample shuttle of limited size and inertia and rely on easily manageable pressures. Yet the high-field landing must be accommodated, possibly by a specially designed probe, which limits the range of applications and, most importantly, the sensitivity (Charlier et al., 2013). In addition, the hard landings of pneumatic sample shuttles can be detrimental to both the hardware and fragile molecular systems. Most motor-operated systems offer the ability to operate from the top, which allows for the use of conventional probes and can reach state-of-the-art sensitivity in mechanical systems (Chou et al., 2016). In addition, the control of the trajectory at all times prevents the occurrence of extreme forces at the end of the sample shuttle path. Yet the use of a belt or rack in some mechanical systems makes the sample shuttle bulky and prevents close access to the sample at a low field. This can be a limitation for applications such as two-field NMR (Cousin et al., 2016a, b; Kadeřávek et al., 2019; Robertson et al., 2023), where the sample shuttle has to move through radiofrequency and gradient coils. The last version of the Redfield shuttle (Redfield, 2012) featured a sample shuttle with small dimensions along the transverse axes with a long stick that coupled the sample to a belt-and-pulley system sitting on top of the high-field magnet. Yet such a design requires very high ceilings in the laboratory and can be a source of large vibrations throughout the magnet.

**Figure 1:**
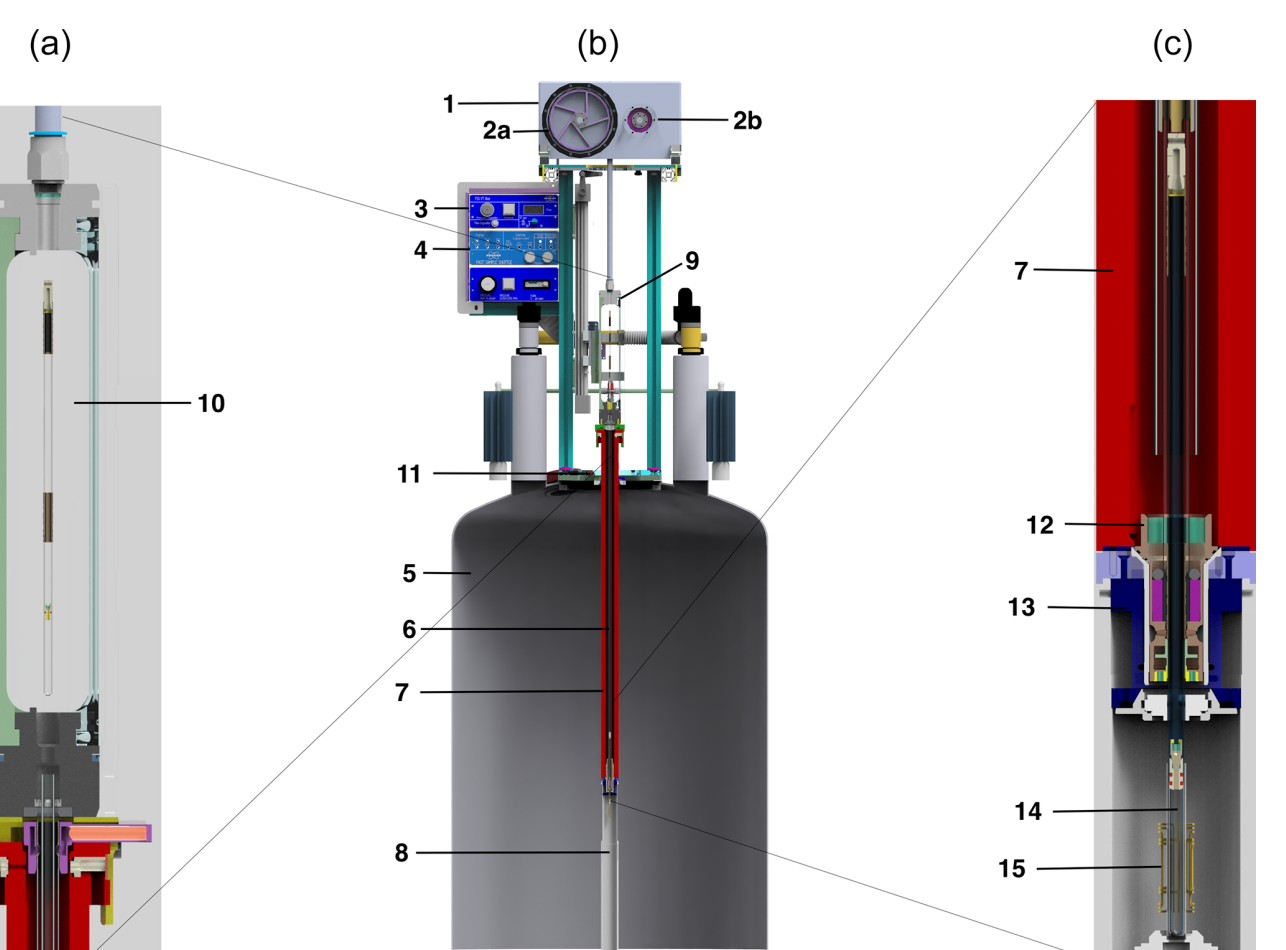
Schematic representation of the fast sample shuttle **(b)**. Details of the sample transfer station **(a)** and the end-stopper assembly at the high-field position **(c)** of the center scheme, respectively. The components presented are the (1) drive unit, (2a) winch wheel, (2b) reversely rotating disk, (3) pneumatic unit, (4) electronic unit, (5) NMR magnet, (6) shuttle guiding tube, (7) shim upper stack, (8) shim system and NMR probe, (9) sample transfer station, (10) window for sample transfer, (11) mounting flange, (12) end stopper, (13) NMR sample turbine, (14) NMR sample container, and (15) NMR RF coil.

Our objective is to develop a new type of sample shuttle system that combines high speed, full control of the trajectory and small transverse dimensions. Here, we introduce a hybrid pneumatic–mechanical sample shuttle system that is able to reach a high speed ( $v_{\max}$ ∼ 27 m s^−1^) with full control of the trajectory (Aders et al., 2022). The principal components of this system are shown in Fig. 1. The diameter of the sample container is d = 6 mm (5 mm for the NMR tube alone), allowing for close access to the sample at low fields for a range of applications. We present the design of this fast sample shuttle and achievable specifications for the motion of the sample shuttle. We illustrate the use of the fast sample shuttle in a broad selection of samples: we compare water proton relaxometry in solutions of paramagnetic ions with fast-field-cycling relaxometry. We measure high-resolution relaxometry of a small molecule that transiently binds to a macromolecule and show the spectral quality and sensitivity of a two-dimensional correlation in a methyl-labeled protein.

## 2 Hardware

The simplest way to move a sample tube out of a vertical high-field NMR magnet is to use a motor and a cord to pull the sample up. Several versions of such a system, from rudimentary to sophisticated designs, have been built in several laboratories (Hall et al., 2020; Miéville et al., 2011; Pileio et al., 2010). As long as the cord is tense, the position of the sample is controlled at all times. The Achilles heel of such a system is the weak acceleration offered by gravity to move the sample back down. Here, we solve this limitation using constant pressure applied from the top of the system. We show a scheme of the principle of this hybrid pneumatic–mechanical system in Fig. 1 and list the characteristic parameters of this fast sample shuttle in Table 1.

### 2.1 Overall design

The fast sample shuttle system includes the following parts, which we describe below:

- a drive unit that includes two 800 W servomotor, a winch wheel and an inertia compensation disk, with a 3.5 bar pressurized gas supply;
- the drive unit controlled by a set of sensors and communicating with the spectrometer using real-time trigger signals and the workstation PC carried by RS232 data exchange;
- a glass shuttle guiding tube, with a dampening system at the high-field end;
- a sample transfer station with an NMR sample container access window;
- a sample container, which consists of a 9 in NMR tube, with two guiding sleeves and an end cap attached to a cord.

**Table 1:** Characteristic parameters of the fast sample shuttle systems as currently installed.

Parameter	Values
Maximum shuttle distance	1020 mm (at 700 MHz), 800 mm (at 600 MHz)
Minimum shuttle distance	50 mm ( > 99.97 % of max field)
Gas pressure	3.5 bar
Gas flow rate range	5 to 20 L min^−1^
Outer diameter of NMR sample tube	5 mm
Maximum shuttle acceleration	1112.6 m s^−2^ (113.5 × g )
Maximum shuttle speed	26.7 m s^−1^
Shuttle travel time (1020 mm)	68 ms (see Fig. 6)
Shuttle travel time (800 mm)	61 ms
Vibrational artifacts in ^1^H NMR spectrum^*^	< 2 % peak to peak

^*^ This value was evaluated on the residual water proton resonance in D_2_O.

The fast sample shuttle (FSS), shown in Fig. 1, is an accessory that can be fitted in any Bruker high-resolution NMR magnet (5). It uses the already available shim upper stack (7) inner bore to accommodate the shuttle guiding tube (6). This guiding tube interfaces at the top with the sample transfer station (9) and with the NMR sample turbine (13) at the bottom. The NMR sample turbine hosts the end-stopper assembly (12). Inside the guiding tube, the NMR sample container (14) glides at very high speeds up and down between the limits imposed by the NMR magnet geometry. Those limits are on one side of the NMR RF coil (15) and inside at the upper part of the NMR probe (8). At this point, the NMR sample container places the sample active volume at the same position as any standard NMR 7 in glass tube. Therefore, the amount of sample needed for high-resolution relaxometry (HRR) experiments is similar to that used for static NMR experiments in conventional 5 mm tubes. The other limit depends on the length of the magnet measured from its center to almost its upper closing lid (11). For instance, for an ultra-shielded 600 MHz magnet, this distance is $d_{\max600}=$ 800 mm with a field $B_{\mathrm{low}}$ = 36.6 mT. Similarly, for an ultrashield 700 MHz magnet, it is $d_{\max700}$ = 1020 mm with a field $B_{\mathrm{low}}=$ 46.6 mT at this position. On the other end, the minimum travel distance is set to, but is not limited to, $d_{\min}=$ 50 mm. This position is at the border of the homogeneous magnetic field plateau with a magnetic field at 99.97 % of the maximum magnetic field at the magnetic center. Shorter travel distances give access to the same magnetic field as that at the center of the magnet (static NMR condition). It is important to note that the FSS is compatible with any high-resolution NMR probe head.

The sample container is introduced inside the magnet through the sample transfer station (9, 10). This process is done by mechanically moving upward a cylindrical PMMA (polymethyl methacrylate) window for the sample transfer (10) that is attached to a sliding mechanism. At the bottom of the sample transfer station, there is an access to the guiding tube. The sliding mechanism for the sample transfer window is set to a rigid supporting structure that in turn is anchored to the mounting flange (11) at the top of the magnet. The drive unit (1), which includes two motors, is located at the top of the supporting structure. The main motor has a winch wheel (2a), and it is in charge of moving the sample container. A secondary motor, which synchronously countermoves with respect to the main motor, contains a reversely rotating disk with the same inertia as the winch wheel (2b). The function of the secondary motor is to minimize mechanical vibrations caused by the movement of the main motor.

**Figure 2:**
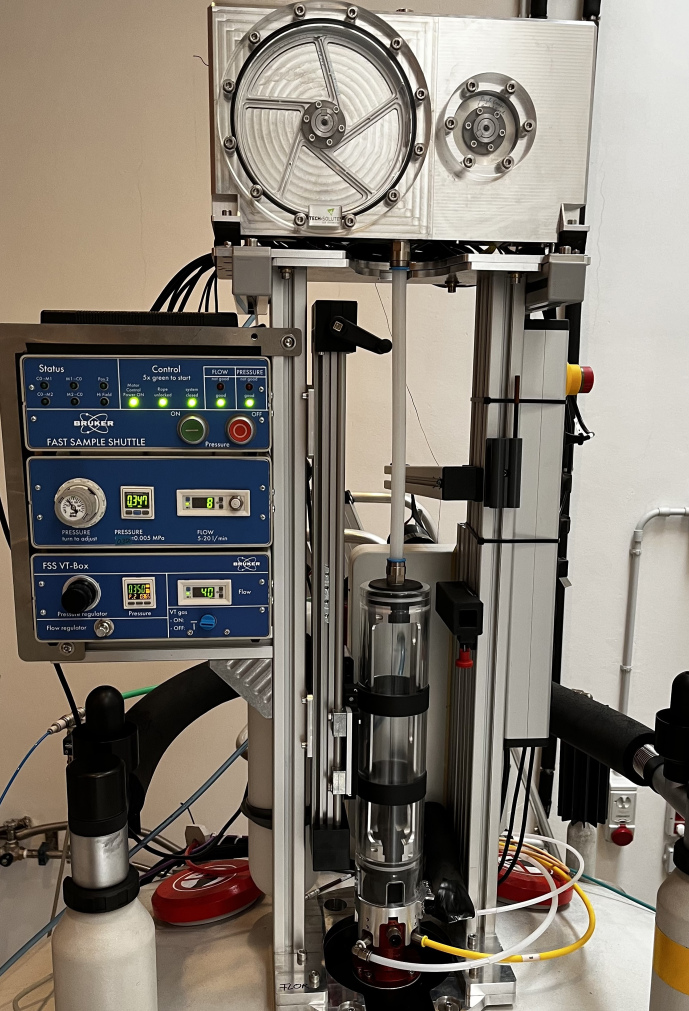
Photograph of the FSS installed on the 700 MHz NMR spectrometer at CERM in Florence, Italy.

To ensure fast deceleration on the way to low fields or efficient acceleration on the way back to the high-field center, gravity is not sufficient. A strong downward force is obtained with air pressure. The pneumatic unit (3) provides the regulated gas pressure and flow. The FSS working pressure is 3.5 bar with a gas flow below 20 L min^−1^. The electronic unit (4) takes care of all FSS safety sensors and provides the communication interface between the motors and the NMR console. In addition, it powers the extension module of each motor as well as the pneumatic unit. Figure 2 is a photograph of the external components of the FSS system built at the Magnetic Resonance Center (CERM).

**Figure 3:**
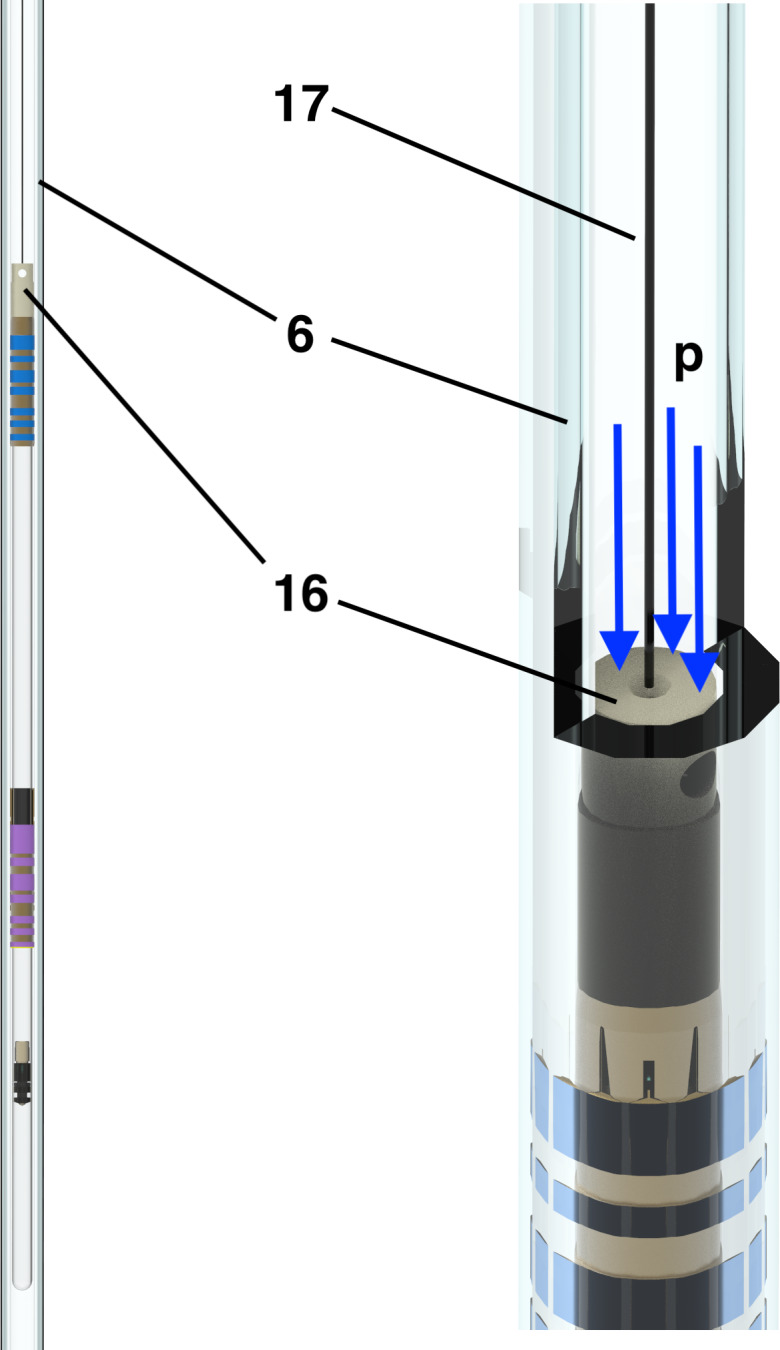
Diagram of sample container in the guidance tube with gas pressure ( p ) applied: (6) shuttle guiding tube (borosilicate 3.3 glass), (16) sample container end cap, and (17) shuttle cord (polyethylene).

The top of the photograph (Fig. 2) shows the two-motor configuration used to compensate for mechanical vibration artifacts. Since the vibrations caused by a single motor and winch wheel are coherent, the second motor and disk in reverse operation is used to cancel out the vibrations of the first. Some vibrations remain after the sample container has landed at the end stopper, requiring a short stabilization delay. After a stabilization delay ( $\tau_{\mathrm{st}}$ = 150 ms), vibration artifacts in proton spectra are less than 2 % of the peak intensity (see below). Figure 3 shows the diagram of the upper part of the sample container (14) under pressure ( p ). In order to ensure full control of the trajectory, the shuttle cord (17) that is connected to the end cap (16) and, in turn, to the sample container (14) must always be under tension during operation. This requires that the force exerted by the pressurized gas be higher than the product of the mass of the sample container and the maximum acceleration. With a top surface area of $s_{\mathrm{top}}$ = 28 mm^2^ and a mass of $m_{\mathrm{container}}=$ 4.7 g, an acceleration of a = 100×g is compensated by a pressure of P ∼ 1.5 bar. The gas pressure chosen for the current design is 3.5 bar. The sample container position is indirectly monitored and precisely controlled by the angular variation of the servo motor (800 W servo motor, JVL MAC800; https://www.jvl.dk/, last access: 24 June 2025).

**Figure 4:**
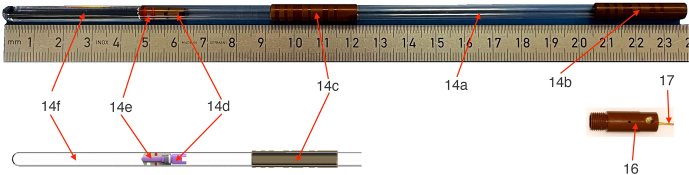
Sample container components. (14a) High-precision thin wall NMR tube with a diameter of d = 5 mm and length of l = 9 in, (14b) upper sleeve with thread, (14c) lower sleeve, (14d, 14e) plug elements to limit the sample volume (14f), and (16) end cap with shuttle cord (17) attached.

Figure 4 depicts in detail the components of the sample container (14). A 9 in long borosilicate 3.3 glass with a 5 mm outer diameter is the base of the sample container (14a). This thin-wall NMR tube has two Vespel™ sleeves, one at the upper end (14b) and another one (14c) positioned to balance the assembly. The sample volume (14f) is about 650 µ L, and it is confined by a two-component seal plug (14d and 14e). The plug allows us to remove trapped air and keeps the sample positioned at the bottom of the tube. The end cap (16) has a small orifice that allows us to keep the sample plug under the working pressure. The inner part of the upper sleeve (14b) of the sample container is threaded so that it can be screwed to the base of the end cap. The cord (17) is connected to the end cap by a knot that tightens upon tension (Patil et al., 2020). The flow of the pressurized gas around the sample container ensures the proper orientation of the sample container in the guiding tube as the bearing gas does for a magic-angle spinning rotor. In addition, the thin layer of gas minimizes contact and hence friction between the sample container and the guiding tube.

**Figure 5:**
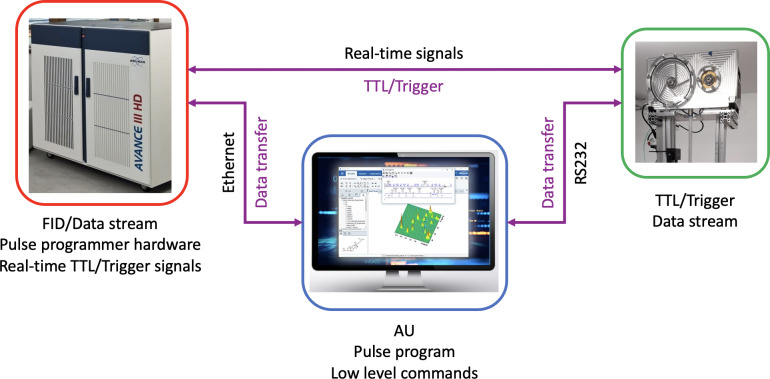
Data communication scheme among the workstation PC, NMR console, and driving motors.

The different communication paths that are needed for the function of the fast sample shuttle are shown in Fig. 5. A control program (AU program) run from the workstation that controls the spectrometer centralizes all communication channels. This AU program tells the motors when and how to move and provides them with relevant information about the position and acceleration as well as several functionalities to simplify the handling of the FSS. This channel of communication uses a bidirectional RS232 serial-port protocol that reads and writes motor registers. In addition, the AU control program calls the NMR pulse sequence and activates real-time signals (transistor–transistor logic, TTL) from the NMR console. Those signals are sent towards the electronic unit (4 in Fig. 1) and transferred to the motors if the operational conditions are met. The operational conditions are monitored by the electronic unit. This unit checks the status of the motors and sample transfer station (closed), the pressure and flow rate values, and whether the shuttle cord is unclamped. When the sample container arrives at the target low-field position, the motors send a trigger signal back to the NMR console, which is then transferred to the workstation via ethernet. At the end of the predefined relaxation delay time at the low-field position, the sample container is sent back to the high-field magnetic center, and the communication process is repeated for the following transient.

### 2.2 Trajectory of the sample container

**Figure 6:**
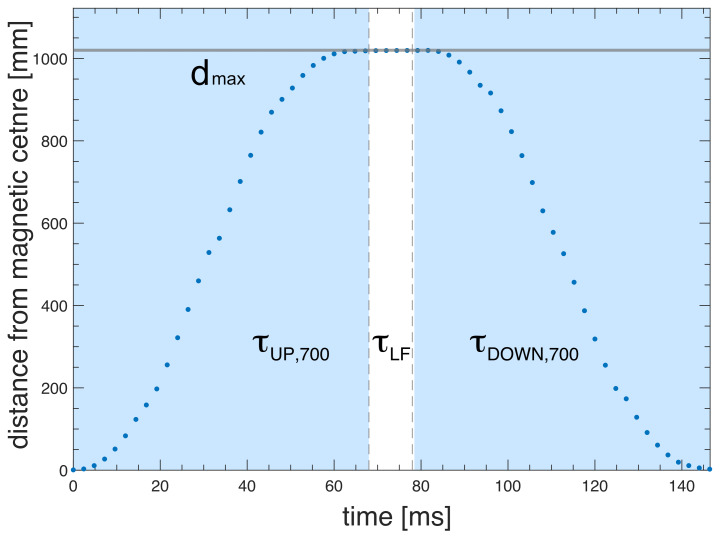
Measured shuttle trajectory (travel distance vs. time) for the FSS installed at the 700 MHz NMR spectrometer at CERM in Florence.

**Figure 7:**
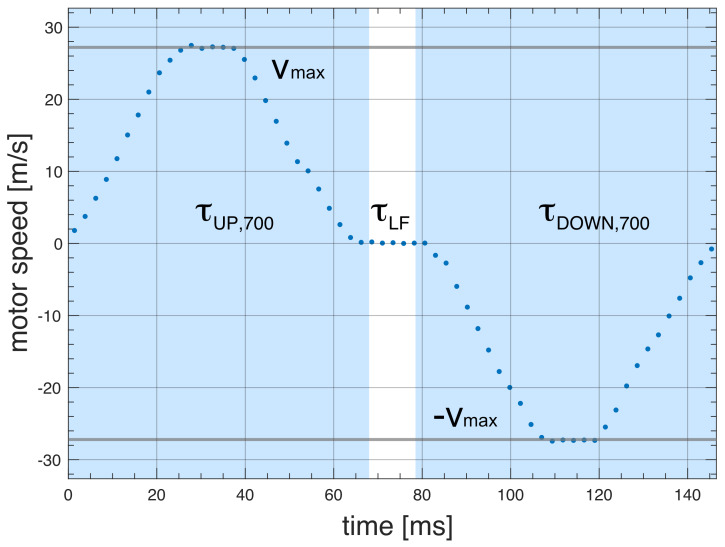
Measured shuttle trajectory (motor speed vs. time) for the FSS installed at the 700 MHz NMR spectrometer at CERM in Florence. The relaxation time at the low field position is $\tau_{\mathrm{LF}}$ .

Full control of the trajectory of the sample container (position as a function of time) is essential for reproducibility and to ensure the acceleration is kept within boundaries tolerable by the sample and spectrometer. The “soft landing” of the sample container at the high-field magnetic center is critical to minimize vibrations and to avoid additional shock loads to the system. As an example, Figs. 6 and 7 show experimental data of the FSS trajectory, as obtained by the ultrashield 700 MHz NMR system at CERM in Florence, for a full shuttle cycle. The motion starts at time zero at the high-field position (sample in the NMR probe) and then moves up 1020 mm to the furthest low-field position within a time $\tau_{\mathrm{UP}}=$ 68 ms (Shuttle UP). The sample resides at the low-field position for a set delay, which is $\tau_{\mathrm{LF}}=$ 10 ms here. Note that the minimal value of the residence time at a low field is $\tau_{\mathrm{LFmin}}=$ 3 ms. Subsequently, the sample is shuttled down back to the high-field position again during $\tau_{\mathrm{DOWN}}=\tau_{\mathrm{UP}}$ . Similar profiles were obtained for the ultrashield 600 MHz NMR systems at École normale supérieure (ENS) and at Bruker. In the latter case, the travel distance is shorter (800 mm), leading to shorter transfer durations of $\tau_{\mathrm{DOWN},600}=\tau_{\mathrm{UP},600}=$ 61 ms. The increment in transfer duration is only 7 ms for an increase in the distance of 220 mm as the extra distance is covered at the highest speed at zero acceleration. The acceleration is constant for a given fixed travel time between zero velocity and maximum velocity (see Figs. 6 and 7). Of course, the sign of acceleration changes depending on whether the shuttle motion is going up or down.

**Figure 8:**
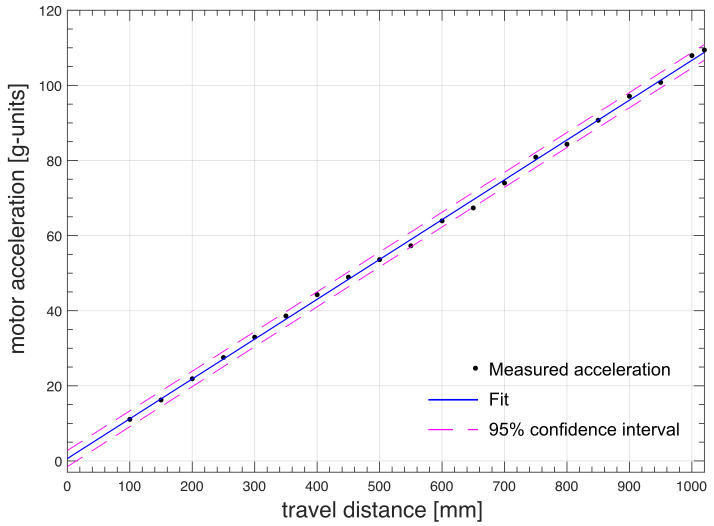
Measured motor acceleration vs. travel distance at constant travel time ( $\tau_{\mathrm{UP},700}=$ 68 ms) for the FSS installed at the 700 MHz NMR spectrometer at CERM in Florence.

The trajectories of the sample container follow the motion with constant acceleration up to the middle of the trajectory or a predefined maximum speed $v_{\max}$ , as experimentally demonstrated in Figs. 6 and 7. Therefore, as long as the maximum speed is reached only for a small fraction of the path, for a given travel time, the motor acceleration is proportional to the travel distance. The minimum travel time was determined to be $\tau_{\mathrm{UP},700}=$ 68 ms for $d_{\max}=$ 1020 mm and $\tau_{\mathrm{UP},600}=$ 61 ms for $d_{\max}=$ 800 mm. By keeping the travel time constant, the motor acceleration was measured for all other distances plotted in Fig. 8. The experimental data were linearly fitted along with a 95 % of the prediction interval of confidence (solid and dashed lines, respectively). Similar results were obtained for the NMR system at ENS in Paris.

**Figure 9:**
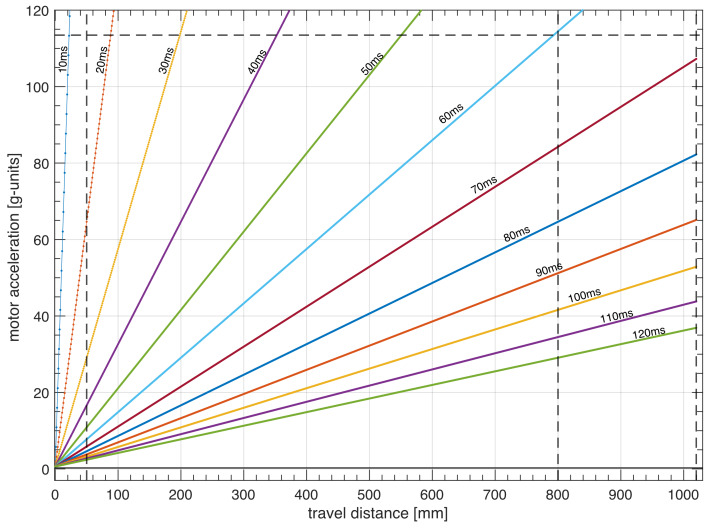
Achievable shuttle travel times as a function of travel distance and motor acceleration.

Polarization losses due to relaxation during transfers between high and low fields should be minimized. Could the delay of $\tau_{\mathrm{UP}}$ and $\tau_{\mathrm{DOWN}}$ be significantly reduced? The transfer delays $\tau_{\mathrm{UP}}=$ 61 ms for a distance of $d_{\max}=$ 800 mm and $\tau_{\mathrm{UP}}=$ 68 ms for a distance of $d_{\max}=$ 1020 mm requires reaching the maximum acceleration achievable $a_{\max}=$ 113×g (Fig. 9). The acceleration increases quadratically with decreasing transfer delays. Reaching slightly shorter transfer times, for instance, $\tau_{\mathrm{UP}}=$ 50 ms for a distance $d_{\max}=$ 800 mm would require an acceleration $a_{\max}=$ 150×g . A much shorter duration of the transfer of the sample shuttle would require accelerations that are impossible to achieve with the current design.

### 2.3 Effects of vibrations on proton spectra

**Figure 10:**
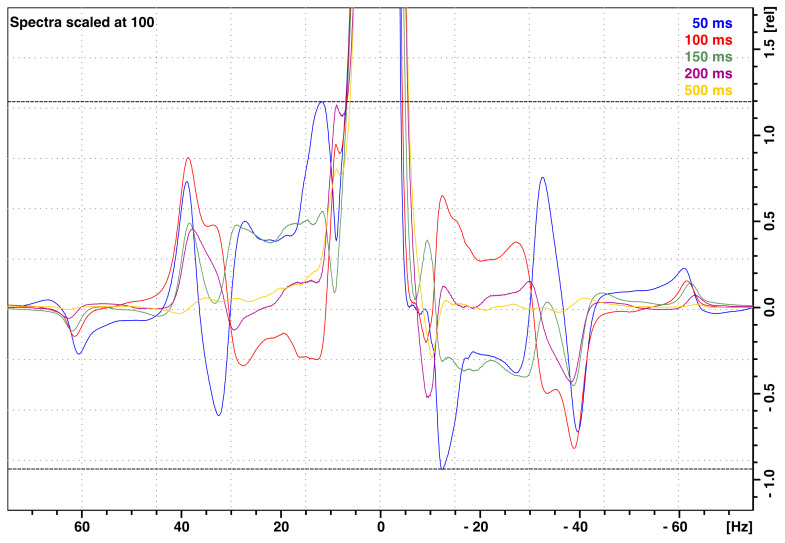
^1^H NMR spectra of 99.8 % D_2_O (residual water peak 0.2 % H_2_O) with the vertical axis in percentages of the peak maximum showing vibrational sideband artifacts at the base of the NMR peak. The various spectra indicate the vibrational artifacts after stabilization times (time interval between shuttle landing and 90° pulse) $\tau_{\mathrm{st}}=$ 50 (blue), 100 (orange), 150 (green), 200 (burgundy), and 500 ms (yellow). Data were taken on the 700 MHz spectrometer installed at CERM in Florence with the maximum 1020 mm travel distance.

The forces generated by the motors (2a and 2b in Fig. 1) lead to vibrations that propagate throughout the Dewar and NMR probe despite the compensation provided through their reverse motion and minimal disturbances of the sample container motion. The motion of the motor is at the origin of the vibration artifacts. These vibrations remain when the shuttle container is static (with no cord) and the motors are moving (see the Supplement). As a consequence, after the landing of the sample shuttle container in the NMR probe, it is necessary to insert a waiting period (stabilization delay $\tau_{\mathrm{st}}$ ) to allow for the decay of residual vibrations. These residual vibrations lead to artifacts in ^1^H NMR spectra in the form of vibrational sidebands, with frequency distances ranging from 7 to 60 Hz around the main NMR line (Fig. 10). On the FSS system installed on the 700 MHz NMR spectrometer available at CERM, the total amplitudes of these sidebands are at most 2.2 % (peak to peak) of the peak height after a stabilization delay as short as $\tau_{\mathrm{st}}=$ 50 ms after shuttle motion from the maximum distance of $d_{\max}=$ 1020 mm. Similar vibration profiles and vibration amplitudes were observed on the FSS system installed on the 600 MHz NMR spectrometer located at Bruker in Wissembourg, France. However, those vibration amplitudes were significantly higher on the FSS system installed on the 600 MHz NMR spectrometer at ENS in Paris in spite of manufacturing with the same tight tolerances for all system components as for the one in Wissembourg.

**Figure 11:**
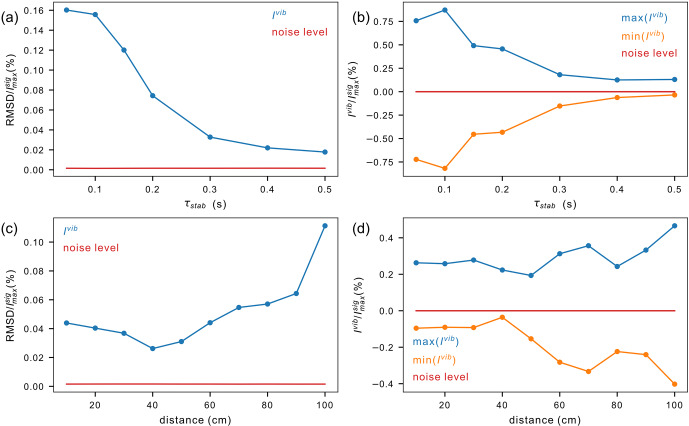
Amplitude of vibration artifacts in ^1^H NMR spectra of 99.8 % D_2_O (residual water peak 0.2 % H_2_O). Vibrations were quantified in the intervals [ - 200, - 20 Hz] and [20, 200 Hz] around the water peak. The level of the noise (red lines) is given as a comparison. **(a–b)** Decay of vibration artifacts after shuttle transfer from the furthest distance ( $d_{\max}=$ 1020 mm) at different stabilization times $\tau_{\mathrm{st}}$ . **(a)** Root mean square deviations of signal amplitudes normalized by the maximum peak intensity. **(b)** Maximum and minimum intensities of vibration artifacts normalized by the maximum peak intensity. **(c–d)** Extent of vibrations 150 ms after shuttle landing as a function of the distance of shuttle displacement **(d)**. **(c)** Root mean square deviations of amplitudes normalized by the maximum peak intensity. **(d)** Maximum and minimum intensities of vibration artifacts normalized by the maximum peak intensity.

A stabilization delay $\tau_{\mathrm{st}}$ is inserted in pulse sequences to reduce vibration artifacts in spectra. This stabilization delay leads to polarization losses due to longitudinal relaxation. How long should the stabilization delay be? Vibration artifacts decay with increasing stabilization delay $\tau_{\mathrm{st}}$ (Fig. 11a–b). With a maximum travel distance of 1020 mm, vibration artifacts are small, with a peak to peak of about 1 % of the peak amplitude after 150 ms, and mostly fade out after 500 ms. As the acceleration is proportional to the travel distance, the forces exerted by the motors on the structure increase with increasing travel distance, leading to an increased level of vibration (Fig. 11c–d). The stabilization time was set to 150 ms, and then the travel distance was varied between 100 and 1020 mm.

**Figure 12:**
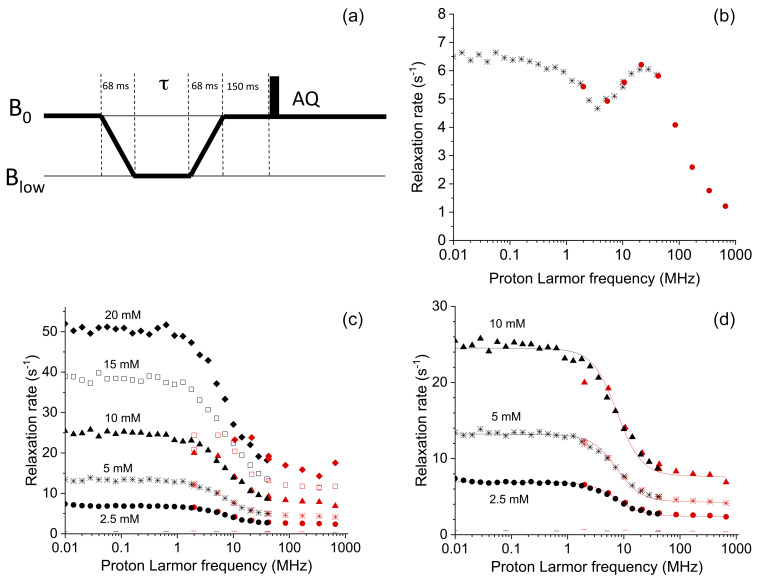
**(a)** Pulse sequence used for the shuttle measurements. The time τ during which the sample is kept at low fields was set to 16 different values ranging from 0 to 1.75 s. **(b)** Field dependence of the longitudinal relaxation rates of water protons in a 0.25 mM solution of Gd-AIE at 15 °C and **(c)** in 2.5, 5, 10, 15, and 20 mM solutions of Cu(NO_3_)_2_ dissolved in a 20 mM HClO_4_ solution of 90 % H_2_O and 10 % D_2_O, at 15 °C; the - symbols indicate the rates of the buffer alone. Black symbols indicate data collected with the Stelar FFC relaxometer (0.01–40 MHz), and red symbols indicate data collected by the Bruker 700 MHz spectrometer equipped with the shuttle system (2–700 MHz). **(d)** Best-fit profiles of the relaxation rates of water protons in 2.5, 5, and 10 mM solutions of Cu^2+^ aqua ion.

### 2.4 Advantages of the design of the fast sample shuttle

During the course of more than 2.5 million shuttling cycles, the system parts show a very low degree of wear and tear. The sample shuttle container and the cord are disposables that can go through about 2 million cycles each. The rate of repetition of shuttling cycles can be high: the test procedure with 2.5 million cycles has been performed with two full cycles per second for about 2 weeks.

The fast sample shuttle is integrated into the TopSpin software and AU programming (see Fig. 5). A single user-friendly interface can be used, and there is no need for external sources to program or control the shuttle units or motors for running relaxometry experiments. Simple instructions have to be inserted in pulse sequences to initiate the shuttle transfers to low fields and back. Any type of pulse sequence and all the features of TopSpin can be used. A full relaxation dispersion profile can be recorded with a single set of instructions. Users create a file listing the target magnetic fields. Either a single list of relaxation delays for all fields or, if necessary, a separate list of relaxation delays can be used for each magnetic field.

Sample filling and sample shuttle container preparation are straightforward, including the positioning of the plugs. In the case of trapped residual air bubbles in the sample volume, the sample shuttle is moved up and down in the shuttle guiding tube to remove the bubbles, making this procedure fast, reliable, and simple.

The position of the sample is accurately calibrated when the shuttle sample container reaches the end stopper (12). When the lower edge of the lower sleeve (14c) touches the soft end stopper, the end stopper exerts an opposite force to that of the working pressure over the container. As a consequence, the torque of the motor decreases. This change in torque is a precise measurement of the position of the sample container. For a given value of the torque, the sample is centered at the high-field position, and the motor count (position) is stored and used for comparison with the next cycle. This process is automatically repeated at every cycle to maintain the same high-field position and compensate for changes in the length of the cord due to temperature variations, humidity, use, etc. This dynamic calibration makes it possible to run experiments for very long periods of time (days to weeks) with no need to interrupt the experiment for calibration of the position.

Once the shuttle system is installed in the NMR magnet, standard NMR experiments are still possible without the deinstallation of the setup. Sealed NMR samples as well as standard 7 in NMR tube samples can be inserted with the help of a crank mechanism, which is attached next to the transfer station and uses fitting holders to manipulate the sample. In addition, the crank mechanism is made in such a way that it is not possible to run shuttle experiments while it is in use. Otherwise, the sample shuttle containers can also be used for static NMR experiments.

**Figure 13:**
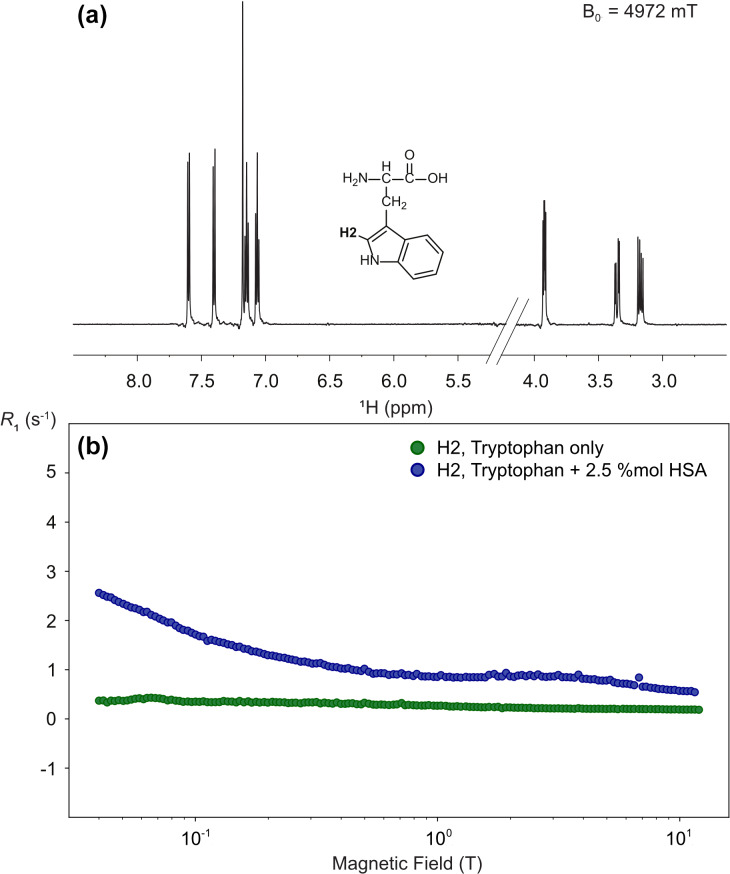
High-resolution proton relaxometry of tryptophan. **(a)** Structure and one-dimensional proton spectrum of tryptophan at 14.1 T obtained after a transfer to a magnetic field $B_{\mathrm{low}}=$ 4.97 T, a relaxation delay $T_{\mathrm{rel}}=$ 3 ms, and a stabilization delay at high-field $\tau_{\mathrm{st}}$ = 150 ms. **(b)** Nuclear magnetic relaxation dispersion profiles for tryptophan proton H2 in the absence (green) and the presence of 2.5 % equivalent of human serum albumin (blue). These experiments were run on a 600 MHz spectrometer equipped with a BBFO double-resonance probe, with a long recycle delay of 5 s. Each spectrum was acquired with 8 scans, and 10 spectra were recorded with different relaxation delays for each magnetic field and relaxation was measured at 150 different magnetic fields, leading to a total experimental time of 25 h for each sample.

## 3 Experiments

All experiments were run on either a 700 MHz Avance NEO spectrometer equipped with a TXI triple-resonance room-temperature probe or on a 600 MHz Avance IIIHD spectrometer equipped with a room-temperature BBFO iProbe double-resonance probe.

**Figure 14:**
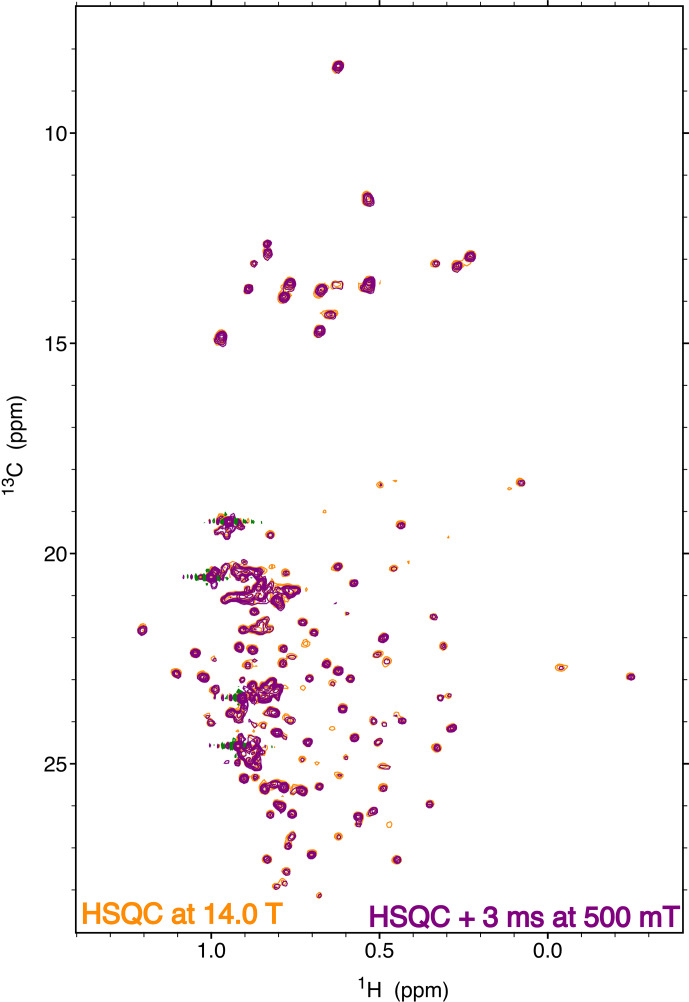
Comparison of a high-field heteronuclear single-quantum coherence (HSQC) spectrum at 14.1 T (orange) and a two-dimensional correlation spectrum obtained at 14.1 T with a transfer to 500 mT (purple). The spectra were recorded on a specifically isoleucine, leucine and valine (ILV)-labeled sample of the protein p 38 γ with ^13^C^1^H^2^H_2_ methyl groups. The HSQC spectrum and the relaxation experiment were both recorded with eight scans and a 3 s recovery delay. Both experiments were run with 128 complex points in the indirect dimension. Each experiment was recorded in just over 2 h. The two spectra are displayed with the same contour level.

### 3.1 Comparison with fast-field-cycling relaxometry

In order to demonstrate the capability of this fast sample shuttle to measure relaxation rates, we compared magnetic-field-dependent relaxation rates measured with the fast sample shuttle and a more conventional approach, fast-field-cycling (FFC) relaxometry. In FFC relaxometry, an electromagnet is used, allowing for fast switching of the magnetic field, typically within a few milliseconds. However, the magnetic field homogeneity is insufficient to be able to record high-resolution spectra. For the sake of comparison, we measured longitudinal relaxation rates of water protons in solutions of a self-aggregating gadolinium(III) complex and of copper(II) aqua ions at different concentrations using both methods. Longitudinal relaxation rates were measured with the fast sample shuttle installed on a 700 MHz spectrometer using a pulse sequence that is equivalent to the “prepolarized” sequence implemented in FFC relaxometry (Fig. 12a). The probe was detuned, and small-angle RF pulses (10°) were applied in order to avoid problems in quantification of the signal intensities due to radiation damping.

The longitudinal relaxation rates of water protons measured for a 0.25 mM solution of a self-aggregating gadolinium complex in water (Gd-AIE) (Li et al., 2019) are shown in Fig. 12b. The agreement between the data measured with the FFC relaxometer and with the shuttle system is excellent in the overlapping range of frequencies. Measurements with the fast sample shuttle permit measuring relaxation rates at fields higher than the FFC relaxometer, covering an additional range of static fields from 1 to 16.5 T.

Relaxation rates measured with the shuttle system for the 2.5, 5, and 10 mM copper(II) solutions (Fig. 12c) are also in excellent agreement with the data measured with the FFC relaxometer in the overlapping range of frequencies (only for the 10 mM sample is the value at 2 MHz somewhat smaller when measured with the shuttle). The fit of these data to the Solomon equation is very good (see Fig. 12d), using as fit parameters only a single correlation time (resulting in 32 ps) and the copper(II)–water proton distance (resulting 2.7 Å, assuming six water molecules coordinated to the copper(II) ion, in fast exchange with bulk water molecules). On the other hand, faster relaxation is not well characterized in the sample shuttle. With 15 and 20 mM copper(II) solutions, longitudinal relaxation rates at magnetic fields below 10 MHz (or 0.25 T) exceed 25 s^−1^ when measured with the FFC relaxometer. When the sample shuttle is used, the longitudinal relaxation decay rates seem to plateau at ∼ 25 s^−1^. The precision of the measurements is also reduced, with errors of about 10 % due to the fact that longitudinal polarization is almost at equilibrium at a low field when the shortest relaxation delay is used. Overall, these data demonstrate that relaxation rates measured with the sample shuttle are in excellent agreement with those measured with conventional relaxometry when in the range from 1 to 20 s^−1^. On the other hand, relaxation rates faster than 20 s^−1^ cannot be measured accurately with the fast sample shuttle apparatus.

### 3.2 Proton high-resolution relaxometry

High-resolution relaxometry is also a sensitive tool to detect the interaction of small molecules with molecular assemblies of larger sizes. Indeed, while the extreme narrowing regime governs most of the nuclear magnetic relaxation dispersion profiles of protons or phosphorus-31 nuclei in a small molecule in solution, a strong dispersion is expected at sub-tesla fields for their relaxation in macromolecules (Pu et al., 2009; Wang et al., 2021). When a small molecule binds transiently to a macromolecule and exchanges between the free and bound states faster than it relaxes, the nuclear magnetic relaxation dispersion is a population weighted average between those expected for the free small molecule and the complex with the macromolecule. Here, we use proton relaxometry of a proton in the indole heterocycle of the amino acid tryptophan to probe the transient binding of tryptophan to human serum albumin. As expected, the nuclear magnetic relaxation dispersion (NMRD) profile of the H2 (or δ 1) proton of tryptophan alone in solution is mostly flat (Fig. 13b) thanks to the absence of vicinal scalar couplings (Miesel et al., 2006). On the other hand, the addition of as little as 2.5 % equivalent of human serum albumin to the solution leads to a measurable dispersion. Proton relaxometry in small molecules is a demanding application of high-resolution relaxometry as narrow lines can be visibly distorted by low-frequency vibrations (Fig. 13a). Here, the presence of such small artifacts does not prevent the measurement of relaxation rates of individual protons.

### 3.3 Sample shuttling of an isotopically labeled protein

The resolution and sensitivity of the fast sample shuttle installed on a conventional high-field NMR spectrometer give access to low-field relaxation of macromolecules. We demonstrate the ability to record high-resolution spectra of a biological macromolecule on a 200 µ M sample of the 42 kDa protein kinase p 38 γ (Fig. 14). The protein was perdeuterated and selectively labeled at the $\delta_{1}$ positions of isoleucine residues, one δ position of leucine residues and one γ position of valine residues with ^13^C^1^H^2^H_2_ methyl groups. A two-dimensional experiment that corresponds to the shortest delay ( $T_{\mathrm{rel}}$ = 3 ms) of a longitudinal carbon-13 relaxation at 500 mT is compared to a high-field heteronuclear single-quantum coherence (HSQC) spectrum (Fig. 14). Both spectra are recorded by the same 600 MHz spectrometer equipped with the fast sample shuttle. The spectral quality of both experiments is identical. A few peaks are significantly attenuated in the relaxometry spectrum due to faster longitudinal relaxation at a low field during the transfer of the sample container between high and low magnetic fields.

## 4 Conclusions

We have introduced a new design of a sample shuttle for applications that combine high-field NMR and an evolution at lower magnetic fields. This sample shuttle is installed on a high-field NMR magnet with a conventional high-field probe, providing state-of-the-art sensitivity and versatile applications. The sample shuttle is a hybrid pneumatic–mechanical design where the upward force is applied to the shuttle tube by a cord, pulled by a motor and winch wheel, and the downward force is applied with moderate (3 to 4.5 bar) overpressure. With an acceleration up to 113.5 × g units, a speed of 26.7 m s^−1^ can be reached in ca. 27 ms. A transfer delay as short as 61 ms can be achieved to reach a magnetic field of 36.6 mT, 800 mm above the magnetic center of a 14.1 T superconducting magnet. Importantly, the sample shuttle is narrow, which will be adapted for magnetic manipulation at a low field in future setups. We demonstrate that low-field longitudinal relaxation rates as high as 20 s^−1^ can be measured in solutions of paramagnetic ions in excellent agreement with fast-field-cycling relaxometry. We also show nuclear magnetic relaxation dispersion profiles of a single proton in the amino acid tryptophan and obtain high-quality spectra of a 42 kDa protein specifically labeled at methyl groups. The fast sample shuttle offers new opportunities for the development of high-resolution relaxometry and other sample shuttle applications in NMR.

## Supplement

10.5194/mr-6-229-2025-supplement The supplement related to this article is available online at https://doi.org/10.5194/mr-6-229-2025-supplement.

## Data Availability

The data presented in Figs. 12, 13, and 14 are available at 10.5281/zenodo.16894282 (Ruda et al., 2025).

## Code availability

The code used to obtain the NMRD profiles from a series of one-dimensional spectra using Nmrglue (Helmus and Jaroniec, 2013) is provided alongside the data (Ruda et al., 2025).
