# Supplementary material for: A fast sample shuttle to couple high and low magnetic fields and applications in high-resolution relaxometry
Source: Magn Reson (Gott). 2025 Sep 3;6(2):229–41. doi: 10.5194/mr-6-229-2025 (PMC12614101; doi:10.5194/mr-6-229-2025)
Supplement: The supplement related to this article is available online at https://doi.org/10.5194/mr-6-229-2025-supplement. [file mr-6-229-2025-supplement.pdf]

Supplement of Magn. Reson., 6, 229–241, 2025
https://doi.org/10.5194/mr-6-229-2025-supplement
© Author(s) 2025. CC BY 4.0 License.

Supplement of

A fast sample shuttle to couple high and low magnetic fields and applications in high-resolution relaxometry

Jorge A. Villanueva-Garibay et al.

Correspondence to: Jorge A. Villanueva-Garibay (jorge.garibay@bruker.com), Fabien Ferrage (fabien.ferrage@ens.psl.eu), and Claudio Luchinat (luchinat@cerm.unifi.it)

The copyright of individual parts of the supplement might differ from the article licence.

S1 Vibration artefacts

Early on in this project, we have determined experimentally that vibration artifacts were still present under the motion of motors, even if the sample were not moving and staying at the high field position, with the cord disconnected from the sample and no pressure applied (see Figure below). These spectra are shown as supporting information.

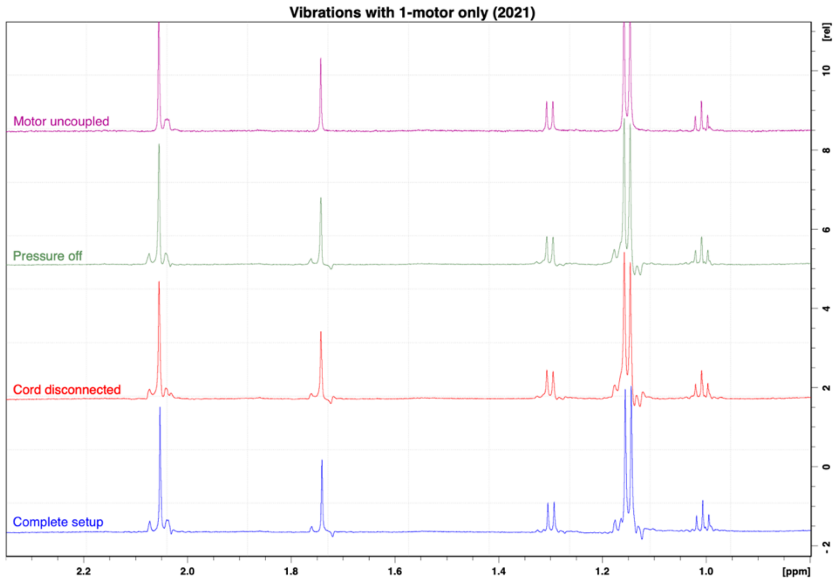

Figure S1. ^{1}H NMR spectra of glucosamine showing mechanical vibration artifacts. (blue) FSS setup with one single motor, it defines the vibration's baseline. (red) The cord is disconnected from the winch wheel and the sample is resting at the high-field position, with pressure and motor still active. (green) Pressure is turned off and transfer station removed, leaving only the motor active. (purple) The motor is uncoupled from the supporting structure: no vibrations are transferred anymore.

S2 Example of pulse program

Below, we give the pulse program used on the Bruker spectrometer to record ^{13}C longitudinal relaxation rates in methyl groups. The commands specific to the use of the shuttle are shown in bold.

```
;Pulse program for High Resolution Relaxometry R1 of methyl carbons in 13CHD2 groups
;For proteins only 13C labeled in methyl groups
;Last change 3/05/2024 LS PHP - remove 13C eq polarisation
;updated to new shuttle, with 2H decoupling, gradients in sensitivity enhancement block
;with decoupling during acquisition
;using f2 - channel for 13C and f4 - channel for 2H
;
;$CLASS=HighRes
;$DIM=2D
;$TYPE=
;$SUBTYPE=
;$COMMENT=


prosol relations=<triple>


#include <Avance.incl>
#include <Delay.incl>
#include <Grad.incl>
```

#include <Shuttle_fss.incl>

```
"p2=p1*2"
"p4=p3*2"
"d11=30m"
"d12=20u"
"d26=1s/(cnst4*4)"


"d0=3u"

"in0=inf2/2"

"DELTA1=d26-p16-d16-4u"
"DELTA2=p16+d16+8u"
"DELTA5=d26-p16-d16-4u"
"DELTA4=d26-p16-d16-p2-d0*2-4u"

#ifdef test
#endif

define list<delay> vd_list = <$VDLIST>

aqseq 312

1 ze
  d11 LOCKDEC_ON
```

```
  d11 LOCKH_ON
  d11 H2_PULSE
  d11 pl12:f2 pl17:f4

2 d11 do:f2 do:f4
  d11 H2_LOCK
  d11 LOCKH_OFF
  ;d12 pl9:f1       ; uncomment next 3 lines and comment out the line after for presat
  ;d1 cw:f1 ph29
  ;4u do:f1
  d1
  50u LOCKH_ON
  d12 H2_PULSE

3 d12 pl1:f1 pl2:f2 pl17:f4
  50u UNBLKGRAMP
  (p3 ph1):f2       ; remove equilibrium carbon polarisation
  4u
  p16:gp0
  d16

  (p1 ph1):f1       ; start of first INEPT Hz -> 2HzCz
  4u
  p16:gp3
  d16
  DELTA1
  (center (p2 ph2):f1 (p4 ph6):f2 )
  4u
  DELTA1
  p16:gp3
  d16
  (p1 ph9):f1
  4u
  p16:gp4
  d16 pl1:f1 pl17:f4
  4u cpd4:f4         ; deuterium decoupling on

  (p3 ph3):f2        ; start refocused INEPT 2HzCz -> Cz
  4u
  p16:gp6
  d16
  DELTA1
  (center (p2 ph2):f1 (p4 ph2):f2 )
  DELTA1
  4u
  p16:gp6
  d16
  (p3 ph10):f2
  3u
  (p1 ph1):f1     ; destroy all 2CzHz terms
  50u do:f4
;  p16:gp7        ; uncomment next two lines for static version
;  d16
```

```
4u BLKGRAMP    ; blank gradient but keep lock hold on
d12
SH_UP          ; macro for shuttle up, comment out for static version
vd_list        ; relaxation
SH_DOWN        ; macro for shuttle down, comment out for static version

d25 pl17:f4    ; stabilization delay at high field
50u UNBLKGRAMP
(p1 ph1):f1    ; destroy all 2CzHz and Hz terms
4u
p16:gp7
d16
4u cpd4:f4     ; deuterium decoupling on
(p3 ph11):f2
4u             ; t1 evolution and transfer Cx ->  a 2CxHz + b 2CyHz
p16:gp2*-1*EA
d16
DELTA5
(p4 ph6):f2
d0
(p2 ph7):f1
d0
p16:gp2*EA
d16
DELTA4
4u do:f4
(center (p1 ph1) (p3 ph4):f2 )      ; beginning of sensitivity enhancement block
4u
p16:gp10
d16
DELTA1
(center (p2 ph2):f1 (p4 ph2):f2 )
DELTA1
4u
p16:gp10
d16
(center (p1 ph2) (p3 ph5):f2 )
4u
p16:gp11
d16
DELTA1
(center (p2 ph2):f1 (p4 ph2):f2 )
DELTA1
4u
p16:gp11
d16
(p1 ph1):f1
DELTA2
(p2 ph1):f1
4u
p16:gp1
```

```
  d16 pl12:f2 pl17:f4
  4u  BLKGRAMP
  go=2 ph31 cpd2:f2 cpd4:f4
  d11 do:f2 do:f4 mc #0 to 2
  F1QF(calclist(vd_list, 1))
  F2EA(calgrad(EA) & calph(ph5, +180), caldel(d0, +in0) & calph(ph6, +180) & calph(ph11, +180) & calph(ph31,
+180))

  d11 H2_LOCK
  d11 LOCKH_OFF
  d11 LOCKDEC_OFF
exit


ph1=0
ph2=1
ph3=0 2
ph4=0 0 2 2
ph5=3 3 1 1
ph6=0
ph7=0 0 2 2
ph8=0
ph9=1 1 1 1 3 3 3 3
ph10={1}*8 {3}*8
ph11={1}*16 {3}*16
ph29=0
ph31=0 2 2 0 2 0 0 2
     2 0 0 2 0 2 2 0
     2 0 0 2 0 2 2 0
     0 2 2 0 2 0 0 2

;pl0 : 0W/120dB
;pl1 : f1 channel - power level for pulse (default)
;pl9 : f1 channel - power level for presat
;pl2 : f2 channel - power level for pulse (default)
;pl12: f2 channel - power level for CPD/BB decoupling
;pl17: f4 channel - power level for CPD/BB decoupling
;p1 : f1 channel -  90 degree high power pulse
;p2 : f1 channel - 180 degree high power pulse
;p16: homospoil/gradient pulse
;p3: f2 channel -  90 degree high power pulse
;p4: f2 channel - 180 degree high power pulse
;d0 : incremented delay (2D)                    [3 usec]
;d1 : relaxation delay; 1-5 * T1
;d11: delay for disk I/O                        [30 msec]
;d12: delay for power switching                 [20 usec]
;d16: delay for homospoil/gradient recovery
;vdlist: low field relaxation delays
;d25: shuttle settle time
;d26 : 1/(4J)YH
;cnst4: = J(YH)
;inf1: 1/SW(X) = 2 * DW(X)
;in0: 1/(2 * SW(X)) = DW(X)
```

;NS: 32 * n
;td1: = td1 for each 2D X l4
;FnMODE: Echo-Antiecho
;cpd2: decoupling according to sequence defined by cpdprg2
;pcpd2: f2 channel - 90 degree pulse for decoupling sequence
;cpd4: decoupling according to sequence defined by cpdprg4
;pcpd4: f4 channel - 90 degree pulse for decoupling sequence
;zgoptns: -DLCKH to deactivate lock commands in Shuttle_fss.incl

;for z-only gradients:
;gpz0: 53%
;gpz1: 20.1%
;gpz2: 40%
;gpz3: 7%
;gpz4: 29%
;gpz6: -17%
;gpz7: 23%
;gpz10: 5%
;gpz11: -11%

;use gradient files:
;gpnam0: SMSQ10.100
;gpnam1: SMSQ10.100
;gpnam2: SMSQ10.100
;gpnam3: SMSQ10.100
;gpnam4: SMSQ10.100
;gpnam6: SMSQ10.100
;gpnam7: SMSQ10.100
;gpnam10: SMSQ10.100
;gpnam11: SMSQ10.100
